# Supplementary material for: Effect of hospital assignment on mortality for AMI patients
Source: BMC Health Serv Res. 2023 Apr 29;23:413. doi: 10.1186/s12913-023-09441-4 (PMC10148466; doi:10.1186/s12913-023-09441-4)
Supplement: Supplementary file 1 — Additional file 1. Appendix. [file 12913_2023_9441_MOESM1_ESM.pdf]

Appendix

Table A1: Instrument validity

	(1) Initially to PCI-hospital b/se	(2) Instrumental value b/se
Age	-0.003***	-0.000
	(0.000)	(0.000)
Male	0.031***	-0.001
	(0.004)	(0.001)
Immigrant	-0.008	0.001
	(0.007)	(0.001)
Cancer	-0.012*	-0.002**
	(0.005)	(0.001)
Diabetes	-0.037***	0.001
	(0.006)	(0.001)
Kidney failure	-0.012	-0.001
	(0.012)	(0.002)
Pulmonary disease	-0.092	0.011
	(0.052)	(0.009)
Cerebro vascular disease	-0.024***	0.000
	(0.007)	(0.002)
Dementia	0.006	0.000
	(0.030)	(0.003)
N	53773	53773
Dep. mean	0.378	0.372
Joint F-statistics	19.75	41.17
Joint P-value	0.000	0.3158

Table A1 note: Column 1 shows estimates of actually being sent initially to a PCI-hospital vs. to a non-PCI-hospital, and column 2 shows the historical municipal share sent initially to a PCI-hospital vs. to a non-PCI-hospital (i.e. the instrument) on observable patient characteristics measured prior to the start of the AMI spell. Both models include fixed effects for year and municipality, and are clustered on municipality. Stars indicate significance level (* p<0.1, ** p<0.05, ***p<0.01)

Table A2: The association between initial admission to a PCI-hospital vs. non-PCI-hospital on mortality at indicated time intervals

	(1)<br>1 month<br>b/se/p	(2)<br>12 months<br>b/se/p	(3)<br>24 months<br>b/se/p
OLS	0.014***	0.002	-0.008
	(0.003)	(0.005)	(0.006)
N	53773	53773	53773
Logit (AME)	0.017***	0.002	-0.010
	(0.004)	(0.007)	(0.008)
N	52827	53241	53443

Table A2 note: The table reports differences in mortality rates for those sent initially to a PCI-hospital compared with those sent initially to a non-PCI-hospital after 1, 12 and 24 months for OLS and Logit models. All models control for age, gender, an interaction between these, year and municipality fixed effect. Regression results from Logit model are reported as the average marginal effect (AME).The sample is slightly smaller in the Logit regressions compared to OLS because of no variation in the mortality variable in some cells. Standard errors (se) are clustered at municipality level. Stars indicates significance levels (* $p<0.1$, ** $p<0.05$, ***$p<0.01$)

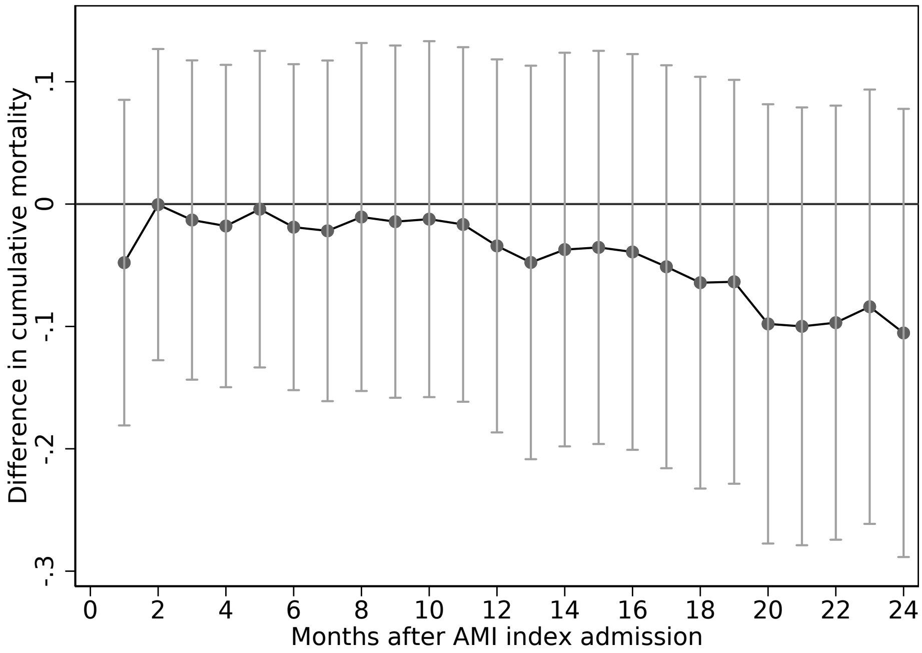

Figure A1: The association between historic share sent directly to a PCI hospital and morality (2SLS)

Figure A1 note: The associations (95% confidence intervals) between the historical municipal share sent directly to a PCI-hospital and mortality for the compliers, in different time intervals. More details on model specification is found in Table 2.

Table A3: The effects of initial admission to PCI-hospital on mortality at indicated time intervals after Acute Heart Attack, estimated with an alternative instrument

	(1)	(3)	(5)
	1 month	12 months	24 months
	b/(se)	b/(se)	b/(se)
ITT(OLS)	-0.003	0.001	-0.005
	(0.009)	(0.012)	(0.012)
IV (2SLS)	-0.020	0.004	-0.041
	(0.072)	(0.098)	(0.096)
FS	0.127***	0.127***	0.127***
	(0.032)	(0.032)	(0.032)
F-test	15	15	15
N	59913	59913	59913

Table A3 note: The alternative instrument equals the share of the ten last AMI patient in a local area that was sent directly to a PCI-hospital. All regressions include controls for age, gender and their interactions, as well as municipality and time fixed effects. Standard errors are clustered at municipality level. Stars indicates significance levels (* $p<0.1$, ** $p<0.05$, ***$p<0.01$)
